# Emerging Families of Ion Channels Involved in Urinary Bladder Nociception

**DOI:** 10.3390/ph3072248

**Published:** 2010-07-19

**Authors:** Isao Araki, Mitsuharu Yoshiyama, Hideki Kobayashi, Tsutomu Mochizuki, Shuqi Du, Yusaku Okada, Masayuki Takeda

**Affiliations:** 1 Department of Urology, University of Yamanashi Interdisciplinary Graduate School of Medicine and Engineering, Chuo, Yamanashi 409-3898, Japan; E-Mails: pxn15164@nifty.ne.jp (M.Y.); hidekik@yamanashi.ac.jp (H.K.); tsutomu@yamanashi.ac.jp (T.M.); matakeda@yamanashi.ac.jp (M.T.); 2 Department of Urology, Shiga University of Medical Science, Otsu, Shiga 520-2192, Japan; E-Mails: iaraki@belle.shiga-med.ac.jp (I.A.); saki@belle.shiga-med.ac.jp (Y.O.); 3 Department of Urology, the 1st Affiliated Hospital, China Medical University, Shenyang, China; E-Mail: du_shuqi@hotmail.com (S.D.)

**Keywords:** ion channel, urinary bladder, sensation, nociception, transient receptor potential

## Abstract

The expression of multiple ion channels and receptors is essential for nociceptors to detect noxious stimuli of a thermal, mechanical or chemical nature. The peripheral sensory transduction systems of the urinary bladder include sensory nerve endings, urothelial cells and others whose location is suitable for transducing mechanical and chemical stimuli. There is an increasing body of evidence implicating the Deg/ENaC and TRP channel families in the control of bladder afferent excitability under physiological and pathological conditions. Pharmacological interventions targeting these ion channels may provide a new strategy for the treatment of pathological bladder sensation and pain.

## 1. Introduction

The lower urinary tract constantly sends mechanosensory information to the central nervous system via the afferent pathway [1]. These signals generate sensation and trigger voiding responses. Pathological conditions alter the chemical and electrical properties of bladder afferent pathways, leading to urinary urgency, increased voiding frequency and pain [1,2]. Chronic conditions that involve tissue inflammation or irritation can induce changes in afferent pathways that lead to hyperalgesia and allodynia. These changes in afferent excitability might be one of pathogeneses for interstitial cystitis/bladder pain syndrome (painful bladder syndrome), which is a chronic pain syndrome of unknown etiology. Patients with the syndrome exhibit urinary frequency, urgency and severe suprapubic pain. Although a variety of treatment regimens have been used to manage bladder pain syndrome, none uniformly eradicate the symptoms of the disease [3]. Peripheral sensitization, lowering of activation thresholds in nociceptor terminals, is a form of stimulus-evoked functional plasticity of afferent pathway [4]. However, the precise mechanisms by which noxious stimuli excite bladder afferents remain unclear.

The sensory specificity of nociceptors is established by expression of multiple ion channels and receptors tuned to respond to particular features of a thermal, mechanical or chemical environment [1,2,4]. The peripheral sensory machinery for nociception in the urinary bladder includes not only sensory nerve endings, but also urothelial cells and others whose location is suitable for transducing mechanical and chemical stimuli. Studies with molecular biology, histochemistry and electrophysiology have revealed that a variety of receptors and ion channels in these peripheral sensory systems are involved in the bladder nociception [2,5,6]. The degenerin/epithelial Na^+^ channel (Deg/ENaC) and transient receptor potential (TRP) channel families are emerging groups of channel proteins implicated in a wide variety of sensory transduction processes in diverse organs [7,8]. In the urinary bladder, there is also an increasing body of evidence for the implication of the Deg/ENaC and TRP channel families in the control of afferent excitability under physiological and pathological conditions [5,6]. Agonists of TRPV1, capsaicin and resiniferatoxin, have been used for therapeutic purposes of bladder overactivity and bladder pain syndrome [9], but their efficacy is limited [10]. 

This review article highlights recent data on the possible roles of the Deg/ENaC and TRP channel families in the pathophysiology of bladder sensation. Pharmacological interventions targeting these ion channels may provide a new strategy for the treatment of pathological bladder sensation and pain.

## 2. Peripheral Sensory Machinery in the Urinary Bladder

**Figure 1:**
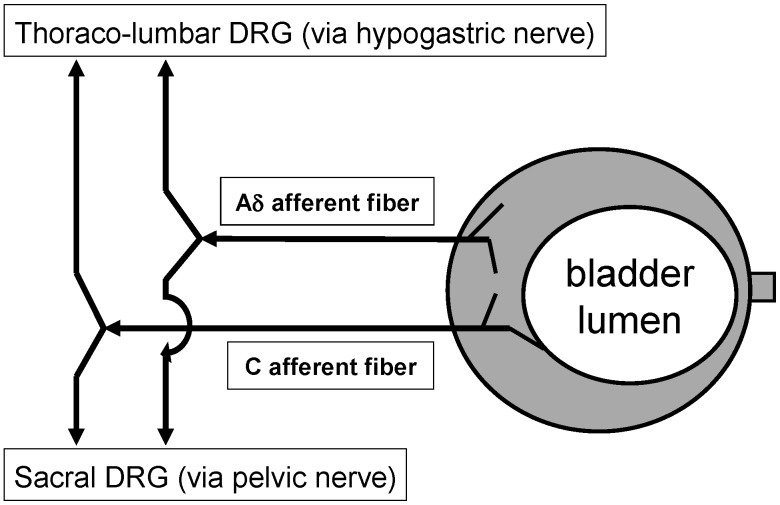
Diagram showing the afferent pathways from the urinary bladder. DRG, dorsal root ganglia.

Sensations from the bladder are conveyed by pelvic and hypogastric nerve afferent fibers (Figure 1) [1]. The pelvic nerves are the principal pathway for afferent input related to micturition. The mechanisms underlying the activation of bladder afferent pathway are not completely understood. 

**Figure 2:**
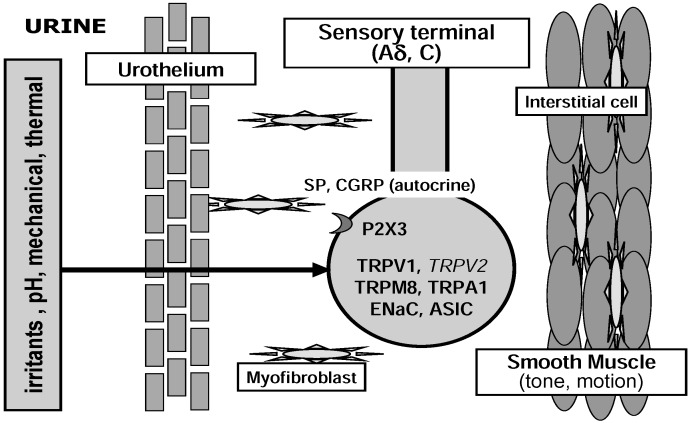
Possible roles of nociceptive ion channels in direct activation of suburothelial sensory nerve terminals. Italic letter indicates ion channels with weak evidence for functional expression.

**Figure 3:**
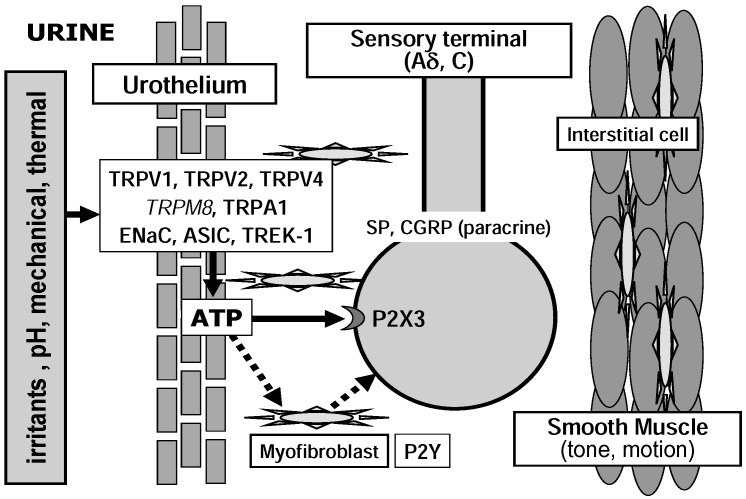
Possible roles of nociceptive ion channels in indirect activation of sensory nerve by mediators released from urothelial cells. Italic letter indicates ion channels with weak evidence for functional expression.

Two main types of mechanisms have been proposed to operate during mechanosensory transduction: indirect chemical and direct physical transduction mechanisms (Figure 2 and Figure 3) [11,12]. An indirect, chemical transduction mechanism relies on activation of afferents by mediators released from non-neuronal cells by mechanical stimulation. A direct, physical transduction is due to direct activation of mechano-gated ion channels in the afferent nerve endings without involvement of extracellular mediators.

### 2.1. Sensory Nerve Endings

The bladder afferent fibers are thinly myelinated or unmyelinated (Aδ and C fibers). In the cat, it has been proposed that Aδ afferent fibers are mechanosensitive and involved in physiological micturition reflex, whereas C-fiber afferents are mechanically insensitive and participate in nociception under painful pathological conditions [13,14]. However, in the rat, both Aδ and C fibers respond to bladder distension [15]. Among stretch-sensitive bladder afferents, low threshold and high threshold afferents have been identified in *in vivo* and *in vitro* preparations. Low threshold fibers are considered to be involved in physiological control of micturition, while high threshold afferents are associated with painful sensations. Both Aδ and C fibers are included in both low and high threshold types. There are no relationship between the conduction velocities of individual mechanoreceptors and their response thresholds [15].

Afferent fibers are abundant within the muscle and in the suburothelial layers [16]. Recent *in vitro* experiments have identified several distinct functional classes of bladder afferents in the rodent [15,17,18]. In addition to the muscle afferents including Aδ and C fibers, the suburothelial nerve plexus primarily comprising C fibers could be mechanosensitive, even under physiological conditions. Zagorodnyuk *et al*. [18] showed that the guinea pig bladder is innervated by at least four classes of extrinsic sensory neurons identified by the location of their receptive fields (mucosal and muscle afferents), their function (mechanoreceptors, chemoreceptors, nociceptors) and the magnitude of their responses (low and high responders). These afferents include stretch-sensitive (1) muscle mechano-afferents and (2) muscle-mucosal mechano-afferents, and stretch-insensitive (3) mucosal mechano-afferents and (4) mucosal chemo-afferents. Muscle-mucosal and mucosal high-responding mechano-afferents have chemosensitivity [18,19]. Recent studies in which bladder afferent responsiveness was assessed without urothelial influence indicated that mechanosensitivity arises by a direct physical mechanism at the nerve endings rather than by a chemical mediator released from the urothelium (Figure 2) [18,19,20]. The density of suburothelial presumptive sensory nerves in the bladder wall is increased in women with idiopathic detrusor overactivity, compared with asymptomatic women [21]. In dorsal root ganglia (DRG), sensory neurons express a variety of receptors and channels including mechanosensitive channels belonging to TRP and Deg/ENaC channel families [22]. At present, however, only a part of these mechanosensitive channels have been demonstrated to be located in the suburothelial nerve plexus (Figure 2). 

Immunocytochemical studies have revealed that numerous peptides, including substance P, neurokinines, calcitonin gene-related peptide (CGRP), vasoactive intestinal polypeptide, enkephalins and cholecystokinin are localized in bladder afferents [23]. Activation of nociceptive ion channels in capsaicin-sensitive sensory terminals induces neuropeptide release from C-fiber terminals [24]. In addition to vasodilatation, extravasation and immune cell migration (neurogenic inflammation), the release of these peptides from sensory nerve endings may be involved in local regulation of sensory nerve excitability, transmitter release from urothelial cells and muscle cell activity [25,26]. In women with idiopathic detrusor overactivity, the densities of CGRP- and substance P-containing nerves are increased in the suburothelial nerve plexus [23].

### 2.2. Urothelium

The bladder urothelium has classically been thought of as a highly effective barrier to ion/solutes. Recently, however, the urothelium has been shown to play an important role in sensory transduction [5,27]. In response to mechanical and chemical stimuli, various neuromediators, such as adenosine triphosphate (ATP), acetylcholine, nitric oxide, prostaglandins and nerve growth factor, are released from urothelial cells [5,27]. ATP is abundant in the cell cytoplasm and can be released extracellularly by several mechanisms, including vesicular exocytosis, transporters such as a member of ATP-binding cassette transporter superfamily, or anion-selective channels such as maxi-anion channel [28]. P2X_3_ is expressed at suburothelial nerve plexus, and exogenous ATP activates several types of bladder afferents and sensitizes their mechanosensory responses [19,29]. Purinergic antagonists reduce distension-induced firing of bladder afferents [30]. Transgenic mice (P2X_3_^-/-^) show reduced urinary bladder reflexes and decreased pelvic afferent firing in response to bladder distension [30]. These findings indicate that ATP released from bladder urothelial cells in response to distention acts on P2X_3_ receptors located in the suburothelial afferent nerve plexus (Figure 3) [5,11,27]. Stretch- or hypotonicity-evoked ATP release from urothelial cells increases in human or feline interstitial cystitis [31,32]. The number of suburothelial P2X3 immunoreactive nerve fibers is increased in the human with neurogenic detrusor overactivity [33], but not in the cat with interstitial cystitis [34]. There is suggestion that receptors for acetylcholine and prostaglandins are also present in bladder afferent endings, although the studies were primarily based on indirect *in vivo* experiments. Muscarinic receptors (M2 and M3) have been supposed to be involved in sensory transduction [35], however those presence is still ambiguous in the suburothelial nerve endings. M2 and M3 immunoreactivity has been shown in the suburothelial nerve bundle [36], while this nerve bundle was located near the detrusor layer and its sensory origin was not defined. However, the reliability of antisera against muscarinic receptors has been recently questioned [37]. Studies using KO mice or an agonist/antagonist suggest that increased production of prostaglandins in the bladder with outlet obstruction sensitizes afferent nerves via EP1 or EP3 [38,39].

It has been shown that urothelial cells express various receptors and channels including receptors for bradykinin (B1 and B2), prostaglandin (EP1 and EP3), neurotrophins (TrkA), purines (P2X and P2Y), noradrenalin (α1 and β3), acetylcholine (muscarinic and nicotinic), pituitary adenylate cyclase-activating peptide, protease-activated receptors and several members of Deg/ENaC and TRP channel families (see below; Figure 2) [5,27]. Activation of these receptors and channels can leads to ATP release from the urothelial cells. These agonist-induced ATP release may increase in cyclophosphamide-induced cystitis [40]. It has been also reported that stretch-evoked ATP release from the urothelium is reduced by blocking α1 adrenoreceptor [41], muscarinic receptors (M2 and M3) [42] or TRP and Deg/ENaC channels (see below). Thus, it is possible that mediators (ATP, Ach, NO and prostaglandins) released from the urothelium modulate ATP release by activating receptors in the urothelium as an autocrine function.

### 2.3. Detrusor Smooth Muscle and Interstitial Cells of Cajal

**Figure 4:**
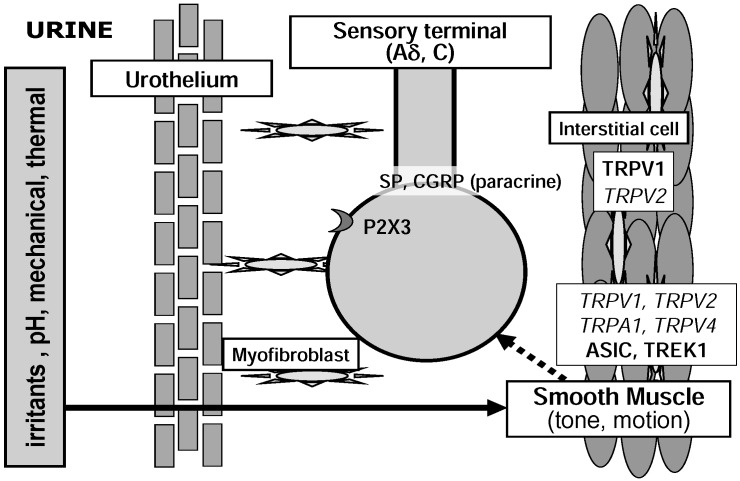
Possible roles of nociceptive ion channels in micromotion and tone of detrusor smooth muscle which might initiate or influence sensory nerve excitation. Italic letter indicates ion channels with weak evidence for functional expression.

Smooth muscles in the bladder show spontaneous contractile activity during the storage phase [43]. These contractions, so called ‘micromotions’, are localized and can be multifocal in separate areas of bladder wall. The autonomous contractile activity in the isolated bladder increases as the bladder is filled. It is still unclear what exactly underlies this spontaneous activity, although the involvements of interstitial cells of Cajal (ICC), intramural ganglia, gap junction and prostanoids are under consideration [43,44]. There is spontaneous and TTX-resistant release of acetylcholine from autonomic nerve endings that affects bladder tone and contractility [45]. It has been suggested that the stretches resulting from local phasic activity of bladder wall generate bladder afferent discharges (Figure 4) [46]. Connexin 43-derived gap junction channels increase in the detrusor muscle from patients with detrusor overactivity or urinary urgency [43]. There is an increase of spontaneous activity in the muscle strips from the human with overactive bladder [47]. 

The bladder has ICC in the detrusor (detrusor ICC), although a human study could not distinguish such cells from fibroblasts [48]. Their functional role in the detrusor is still unexplained, but similar cells generate pacemaker activity responsible for phasic or tonic muscular contraction in the gut [49] and possibly in the urethra [50]. Overactive human detrusors have larger number of c-kit positive detrusor ICC-like cells than control samples [51]. In the detrusor, however, only a small percentage of ICC show spontaneous Ca^2+^ transients, and the frequency and duration of these transients are quite different from those in the smooth muscle [43,52]. Detrusor ICC may modulate the spread of action potentials along the muscle bundles rather than being the pacemaker of spontaneous activity. 

It has been proposed that myofibroblasts beneath the urothelium, which is also called the ICC in the lamina propria (ICC-LP), act as a relay or an amplifier in the sensory response to bladder-wall stretch. Isolated myofibroblasts display spontaneous transients of membrane potential and intracellular Ca^2+^, and response to exogenous application of ATP via P2Y receptor [44,53]. Additionally, muscarinic receptors (M2 and M3) are expressed in presumptive myofibroblasts, although application of the cholinergic agonist, carbachol failed to evoke a response. Suburothelial myofibroblasts have rich connexin 43 gap junctions between adjacent cells and show a close apposition to unmyelinated nerve fibers. Connexin 43 immunoreactivity is increased in the suburothelium of the human with detrusor overactivity, while c-kit immunoreactivity does not [54]. These findings suggest that myofibroblasts link the urothelial ATP signaling to afferent excitation (Figure 3) [44,53]. However, this possibility is unwarranted at present because a functional role of bladder myofibroblasts has not yet been demonstrated.

## 3. Nociceptive Ion Channels in the Urinary Bladder

### 3.1. Degenerin/Epithelial Na^+^ Channel (Deg/ENaC) Family

The Deg/ENaC family represents a new class of cation channels that was discovered at the early 1990s [8,22]. This cationic channel family is characterized by amiloride-sensitivity, and is either constitutively active or activated by mechanical stimuli, and/or by ligands such as peptides or protons. Recent studies on these channels have implicated them in various sensory modalities, such as baroreceptors and cutaneous sensory structures. 

#### 3.1.1. Epithelial Na^+^ Channel (ENaC)

It has been reported that the ENaC is expressed in the mammalian urinary tract epithelia (renal pelvis, ureter and urinary bladder) [55,56,57,58] and suburothelial nerve fibers (unpublished data) (Figure 2 and Figure 3). In rabbit bladder urothelium, ENaC is mechanosensitive, having the ability to change their sodium transport properties following changes in hydrostatic pressure. The basal ATP release from the rabbit bladder urothelium is altered by amiloride, a blocker of ENaC [59]. In cultured urothelial cells, amiloride and Gd^3+^, a non-specific blocker of mechanosensitive channels, suppresses ATP release by a hypotonic stimulus [32]. Furthermore, intravesical infusion of amiloride reduces the frequency of reflex voiding during bladder filling in anesthetized rats, and stretch-evoked ATP release from bladder strips is largely diminished by amiloride [58]. These indicate that ENaC expressed in the bladder urothelium is implicated in the mechanosensory transduction by controlling stretch-evoked ATP release (Figure 3). The ENaC expression in the bladder urothelium has a remarkable species difference between the rat and human [57,58]. In the human bladder, the expression level of ENaC is extremely low, but is markedly up-regulated in obstructed bladders [57]. The expression level of ENaC mRNA correlates significantly with storage urinary symptoms. The over-expression of ENaC in the human obstructed bladder might be associated with the induction of detrusor overactivity by bladder outlet obstruction (BOO).

#### 3.1.2. Acid-Sensing Ion Channel (ASIC)

ASICs, an H^+^-gated subgroup of the Deg/ENaC family, are encoded by three different subunit genes, ASIC1, ASIC2 and ASIC3, and the subunits form homo- and hetero-multimeric channels, which differ in their pH sensitivity and other pharmacological properties [8]. In the central and peripheral nervous system, ASICs have emerged as key receptors for extracellular protons, and recent studies suggest diverse roles for these channels in the physiology of mechanosensation and the pathophysiology of acid-evoked pain [60]. ASICs, especially ASIC1 and ASIC2, are abundantly expressed in the urothelium and detrusor muscle of mouse bladder [61]. ASIC1 is a dominant subunit in the bladder mucosa, and both ASIC1 and ASIC2 are expressed in the bladder muscle. ASIC2 and ASIC3 are expressed in suburothelial nerve plexus of the rat (Figure 2,Figure 3,Figure 4). The expressions of ASIC2 and ASIC3 in the urothelium and suburothelial nerve plexus increase in cyclophospamide-induced cystitis, while ASIC1 expression is not altered [62]. Recent studies in the rat suggested that acid-induced Ca^2+^ influx and ATP release in the urothelium are partly attributed to ASIC activation [63,64]. Roles of each ASIC subunit in sensory function are not well understood at present. The experiments on gastrointestinal sensation indicate that the disruption of ASIC1 or ASIC3 increases or decreases the mechanical sensitivity, respectively, while disrupting ASIC2 has varied effects [65,66].

### 3.2. TRP Channel Family

TRP superfamily is a specific class of cationic channels that gate in response to a diverse array of chemical and physical stimuli [67]. TRP channels have emerged as potent candidates for thermo-, chemo- and mechanosensors in various sensory modalities, although it is controversial in many instances whether TRP channels are directly activated by mechanical stimuli or part of a down stream signaling pathway [68].

#### 3.2.1. TRPV1

TRPV1 is primarily expressed in small to medium-sized primary sensory neurons, the majority of which synthesize neuropeptides such as substance P and CGRP [69]. In the mammalian urinary bladder, the structures in which TRPV1 (vanilloid receptor type 1: VR-1) is expressed include the bladder sensory fibers, urothelial cells, ICC-LP (myofibroblasts) and probably smooth muscle of the rodent and human (Figure 2,Figure 3,Figure 4) [69,70]. However, evidence of TRPV1 expression in non-neuronal cells has been recently questioned with the observation of nonspecific cellular TRPV1-immunoreactivity in bladders from TRPV1 knockout mice [71]. It is almost impossible that nonspecific staining by TRPV1 antibodies against different epitopes is from splice variants. Thus, a part of older reports should be interpreted with care. TRPV1 regulates pain perception and bladder reflex by modulating sensory activity. Agonists of TRPV1, capsaicin and resiniferatoxin, have been used for therapeutic purposes of bladder overactivity and interstitial cystitis [72], but their efficacy is limited [10]. 

TRPV1-immunoreactive nerve fibers form varicose plexuses in the subepithelial layer and the surface of the smooth muscle of the bladder wall [69]. A role of TRPV1 is well established in nociception as an integrator for thermal and chemical noxious stimuli, although acidic stimuli are capable of sensitizing TRPV1-independent mechanisms of bladder sensation [9,73]. Activation of TRPV1 in peripheral nerve endings promotes the depolarization and the release of neuropeptides, such as substance P and CGRP (Figure 2) [74,75]. Capsazepine, a TRPV1 blocker, decreases the frequency of reflex voiding in cyclophosphamide inflamed rat bladder [76]. Cyclophospamide- or acrolein-induced cystitis leads to bladder mechanical hyperactivity in wild-type mice, but not in TRPV1 KO mice [77]. After bladder inflammation with lipopolysaccharide, the frequency of reflex voiding and the number of *c-fos* expressing spinal neurons by innocuous bladder distension increase in mice. These changes in the inflamed bladder are suppressed by a potent TRPV1 antagonist and not observed in the TRPV1-deficient mice [78]. Recently a growing body of evidence has led to the emergence of TRPV1 as a prominent participator in normal sensory transduction. Compared with wild-type, mice lacking TRPV1 has been shown to increase the frequency of non-voiding bladder contraction and enhance bladder capacity under anesthesia [9]. In TRPV1-deficient mice, sensitivity of low threshold bladder afferents to distension is reduced [79] and spinal *c-fos* response to distension is abolished [9]. These results suggest that TRPV1 is implicated in mechanosensitivity of the bladder. However, TRPV1 is not considered to be mechanically gated and a TRPV1 antagonist, capsazepine, had no effect on bladder reflex activity of normal mice [76]. Stroking-induced activity of mucosal high-responding afferents in the guinea-pig is not influenced by capsazepine *in vitro* [20]. 

TRPV1 may be also functional in the urothelium (Figure 3). In excised bladder strips and cultured urothelial cells from mice lacking TRPV1, hypoosmolality-evoked ATP and NO releases are diminished [9]. Calcium influx and ATP release increase in human urothelial cells when exposed to vanilloid compounds [80]. However, recent studies questioned the functional expression of TRPV1 in the mouse and guinea-pig urothelial cells [81,82,83]. Capsaicin induced neither Ca^2+^ influx nor current in cultured urothelial cells [81,82]. The functional meaning of TRPV1 in ICC and smooth muscle is completely unclear at the moment, even if the immunolabelling is specific [71].

#### 3.2.2. TRPM8

TRPM8, a receptor that detects temperature in the “cool” range (<28 °C), expressed in the urothelium and suburothelial sensory fiber might be implicated in the bladder cooling reflex (Figure 2 and Figure 3) [84,85]. Intravesical infusion of menthol, a widely used TRPM8 activator, facilitates the micturition reflex in conscious rats [85]. Intravenous administration of a TRPM8 channel blocker decreases the frequency of isovolumetric bladder contractions without reducing the contraction amplitude in anesthetized rats [86]. These findings suggest that TRPM8 play a role in the bladder afferent pathways (Figure 2). The number of TRPM8-immunoreactive suburothelial nerves is increased in patients with idiopathic detrusor overactivity, and the relative density of TRPM8-immunoreactive nerve fibers significantly correlates with symptoms of overactive bladder and painful bladder syndrome [84]. However, there is a report showing that TRPM8-immunoreactivity is detected in only a small proportion of the DRG sensory neurons innervating the urinary bladder (1.2 %) [87]. Furthermore, TRPM8-deficient mice still have menthol-sensitive neurons [88] and menthol may cause TRPM8-independent release of Ca^2+^ from intracellular stores [89]. Also, in the rat and mouse urothelium, the expression level of TRPM8 is much lower than those of other TRP channels [61,83,90]. The expression of TRPM8 mRNA is not changed in the human bladder mucosa with BOO and detrusor overactivity [90]. TRPM8 does not show any mechanosensitivity originally and its endogenous activators await further studies.

#### 3.2.3. TRPA1

Recently, it was reported that TRPA1 are expressed in the rodent and human bladders [90,91,92,93]. TRPA1 is a potential candidate for mechanosensor and/or nociceptor responding to chemical and thermal stimuli [7]. TRPA1 shares similar channel properties with the mechanosensitive channels of the hair cells. In the DRG and trigeminal ganglia, TRPA1 is expressed in small sensory neurons (C or Aδ) with nociceptive markers, CGRP and substance P, and coexpressed with TRPV1 [94]. TRPA1 agonist(s) causes nociceptive reaction and heat hyperalgesia in the skin [95]. TRPA1 knockout mice displayed behavioral deficits in response to mechanical, cold and chemical stimuli [96], while a conflicting observation was made in another independently developed knockout mice [97]. TRPA1 has been reported to be expressed in the urothelium, suburothelial sensory C-fibers and probably muscle layer of the urinary bladder (Figure 2,Figure 3,Figure 4) [90,91,92,93]. Nagata *et al*. [91] first reported that TRPA1 is expressed at sensory nerve terminals beneath the mouse urinary bladder mucosa. TRPA1 agonists cause a concentration-dependent contraction of isolated rat bladder strips through sensory fiber stimulation [98]. Intravesical infusion of TRPA1 agonists causes bladder hyper-reflexia through C-fiber afferent pathway during continuous infusion cystometrograms in rats [92,93]. In the patient with BOO, the expression of TRPA1 is upregulated in the bladder mucosa [90]. Our preliminary study indicates that TRPA1 is the most abundant TRP channels expressed in the human bladder mucosa (unpublished data). Thus, TRPA1 in the bladder urothelium and/or sensory nerve endings might be involved in the bladder sensory transduction and the induction process of overactive bladder by BOO (Figure 2).

#### 3.2.4. TRPV4

TRPV4 is originally postulated to serve as a mechano- or osmosensor [7]. Recent studies using mice lacking TRPV4 revealed the involvement of TRPV4 in sensing mechanical pressure, osmolality, and warmth *in vivo* [99,100]. TRPV4 is abundantly expressed in the rodent and human bladder urothelium [101,102] and the most abundant TRP channel expressed in the mouse cultured urothelial cells (Figure 3) [83,103]. TRPV4 knockout mice manifest an incontinence phenotype in spontaneous voiding pattern and exhibit a lower frequency of voiding contraction in continuous filling cystometry under anesthesia [101,104]. Intravesical infusion of a TRPV4 agonist induces bladder overactivity in the conscious mice, an effect that is undetected in TRPV4-deficient mice [104]. In rodent cultured urothelial cells, TRPV4 agonist promotes Ca^2+^ influx and current [81,82,83,103], and enhances ATP release [102]. In cultured urothelial cells from TRPV4-deficient mice, stretch-evoked Ca^2+^ influx and ATP release decrease [103]. These findings indicate a critical role of TRPV4 in physiological bladder function and mechanosensory transduction (Figure 2), although recent studies suggest that TRPV4 lies downstream of a mechanosensor and mediates the transduction of mechanical stimuli [68].

A study showed the expression of TRPV4 channel in the mouse bladder smooth muscle and an agonist-induced contraction of bladder strips [104], while other did not observe the alteration of rat bladder strip contractility by a widely-used agonist, 4α-phorbol-12,13-didecanoate [102].

#### 3.2.5. TRPV2

TRPV2 mediates hypotonic swelling and stretch-induced Ca^2+^ influx [105]. TRPV2 has been reported to express in the urothelium, smooth muscle and suburothelial sensory nerve endings of the rodent bladder [83,106,107] and the urothelium of the human bladder (Figure 2,Figure 3,Figure 4) [108]. A known agonist for TRPV2, tetrahydrocannabinol, induced Ca^2+^ influx and current in mouse urothelial cells [83]. A role of TRPV2 in bladder sensory function is yet to be explored. 

### 3.3. Potassium Channels

Many types of potassium channels have been identified in urinary bladder myocytes and thought to be important in controlling membrane potential and excitability [109]. Opening of potassium channels leads to an increase in potassium efflux from cell and produces a hyperpolarization of the membrane potential, thereby relaxing bladder. A number of ATP-activated K^+^ and Ca^2+^-activated K^+^ (BK or maxi-K and SK) channel openers have demonstrated that activation of potassium channels can reduce the spontaneous or stimulus-induced contractions of isolated bladder strips [110]. Some of K^+^ channel openers increase bladder capacity without affecting maximum bladder contraction pressure in accordance with decreasing afferent nerve activity [111,112,113]. In the human, the expression levels of BK channel subunits are reduced in both the smooth muscle and mucosa of obstructed bladder and negatively correlated with lower urinary tract symptoms [114]. The down-regulation of BK channel expression in BOO patients might be involved in the induction mechanisms of OAB by BOO. It may be noteworthy that stretch-activated BK channels (SAKCa) are cloned from chick ventricular myocytes [115]. These data suggest the potential utility of these K^+^ channels for the treatment of overactive bladder. Unfortunately, however, these K^+^ channels exhibit only partial or no bladder selectivity.

The two-pore-domain K^+^ channels (K2P or KCNK) are highly expressed in the central and peripheral nervous system and non-neuronal tissues, and they provide a wide variety of important functions including responses to mechanical stretch, temperature and pH [116]. TREK-1 (KCNK2), a mechanosensitive subfamily of K2P channels, has a higher expression level (12-fold) in the human bladder myocytes relative to the aorta (Figure 4) [117]. Its opener produces a relaxation of KCl-induced contraction in rat bladder strips, but no effect on aortic strips. The expression of TREK-1 protein is decreased in the smooth muscle of mouse bladder with BOO [118]. Systemic administration of a TREK-1 blocker induces an increase in premature detrusor contraction (non-voiding contraction) during bladder filling in anesthetized mice [118]. Our preliminary study indicates that TREK-1 is also expressed in the bladder urothelium (Figure 3) (unpublished data). 

## 4. Conclusions

Sensory transduction may be mediated by a number of factors that together enhance the electrical activity to excite afferents. In response to thermal, mechanical or chemical stimuli, several classes of ion channels in the peripheral sensory machinery are involved in the bladder nociception directly and indirectly (Figure 2,Figure 3,Figure 4). In addition to the stimulus nature to respond, the threshold for activation may be different between distinct ion channels, as recent studies suggested [103]. Thus, it is essential for perceiving multiple forms of stimuli that diverse classes of ion channels with a variety of response characters are expressed. The response characteristics of each ion channel should be explored in the future experiments.

The peripheral sensory transduction systems of the bladder include sensory nerve endings, urothelial cells and detrusor smooth muscle cells. Recent studies assessing bladder afferent sensitivity without urothelial influence suggest that bladder mechanosensitivity arises by a direct physical mechanism at the nerve endings rather than via release of mediators from the urothelium. However, it is still unclear that activation of ion channels at sensory nerve endings leads to direct afferent excitation. A critical sensory transduction pathway may be different under normal and pathological conditions, although these pathways have influence upon each other. Nociceptive ion channels are implicated in neuropeptide release from afferent nerve endings and in mediator release from urothelial cells for autocrine/paracrine signaling. Ion channels may also have a role in the regulation of smooth muscle tone and micromotion. These actions of ion channels in the urothelium and the smooth muscle may contribute to sensory transduction by tuning the excitability of bladder afferents. Pharmacological interventions targeting ion channels in the peripheral sensory machinery may provide a new strategy for the treatment of bladder dysfunction.
